# Prognostic significance of IL-33 and ST2 expression in head and neck squamous cell carcinoma: a systematic review

**DOI:** 10.3389/froh.2025.1551781

**Published:** 2025-03-24

**Authors:** Swetha Acharya, Usha Hegde, Anirudh Balakrishna Acharya, SubbaRao V. Madhunapantula, Huchanahalli Sheshanna Sreeshyla, Priyanka Nitin, Medha Karnik

**Affiliations:** ^1^ Department of Oral Pathology and Microbiology, JSS Dental College and Hospital, JSS Academy of Higher Education and Research (JSSAHER), Mysore, India; ^2^ Department of Restorative Dentistry, College of Dental Medicine, University of Sharjah, Sharjah, United Arab Emirates; ^3^ Center of Excellence in Molecular Biology and Regenerative Medicine (CEMR) Laboratory (DST-FIST Supported Center and ICMR Collaborating Center of Excellence- ICMR-CCoE), Department of Biochemistry, JSS Medical College & Hospital, JSSAHER, Mysore, India

**Keywords:** IL-33, ST2, carcinoma associated fibroblasts, regulatory T cells, head and neck squamous cell carcinoma, prognosis

## Abstract

**Background:**

Interleukin-33 (IL-33) and Suppression of tumorigenicity 2 (ST2) expression are strongly associated with tumor growth and progression in diverse cancers, indicating the possibility of targeting the IL-33/ST2 axis pathway as a favorable therapeutic approach. However, the specific implications of IL-33/ST2 expression in Head and Neck Squamous Cell Carcinoma (HNSCC) prognosis are not fully understood. Thus, there is a need for more comprehensive research to verify the tasks and clinical significance of IL-33 and ST2 in HNSCC.

**Objectives:**

The objective of this study was to evaluate the potential of differentially expressed IL-33 and ST2 in tumor tissues that could serve as novel biomarkers in HNSCC.

**Material & methods:**

The Web of Science, Scopus, and PubMed electronic databases were searched and analyzed from January 2013 to July 2023.

**Results:**

Nine studies fulfilling the inclusion criteria were analyzed. These selected studies were mainly having observational analytical study design, predominantly conducted within the Southeast Asian population. IL-33, primarily located in the stroma, demonstrates enhanced expression within carcinoma-associated fibroblasts (CAFs). Overexpression of IL-33 in CAFs correlates with its expression in tumor cells, as per some of these reports. Elevated IL-33 levels in CAFs are associated with unfavorable clinical outcomes. Increased IL-33 expression is related to poor nodal metastasis-free survival, indicating an adverse prognosis in HNSCC. In HNSCC, tumor cells and regulatory T cells (Tregs) expressed ST2. The degree of ST2 expression on Tregs corresponds to the abundance of IL-33 expressing CAFs. IL-33 increases the Tregs density and amplifies their suppressive capability. Poorer survival outcomes in HNSCC are linked to elevated ST2 expression in Tregs combined with the existence of IL-33-expressing CAFs.

**Conclusion:**

CAF-driven cancer invasiveness relies on IL-33 signaling via paracrine and autocrine pathways. IL-33 may be a prognostic biomarker and therapeutic target, aiming to improve prognosis and survival in HNSCC. The IL-33/ST2 axis significantly configures the tumor microenvironment and tumor aggressiveness in HNSCC. The role of serum IL33 and ST2 remains to be further studied in HNSCC.

**Systematic Review Registration:**

https://www.crd.york.ac.uk/PROSPERO/i, identifier (CRD42023447963).

## Background

Head and neck squamous cell carcinoma (HNSCC) is a group of cancers affecting the oral cavity, pharynx, hypopharynx, larynx, nasal cavity, and salivary glands, making it the seventh most common cancer globally (1). As per the latest GLOBOCAN estimates (2022), HNSCC is accounting for an estimated 946,456 new cases (roughly 4.7% of all cancer diagnoses around the world) and 482,001 deaths per year (roughly 4.9% of global cancer deaths). The incidence includes approximately 389,485 cases of cancer of the lip and oral cavity, 188,960 of the larynx, 120,416 of the nasopharynx, 106,316 of the oropharynx, 86,276 of the hypopharynx, and 55,003 of the salivary glands (2). Its incidence is steadily rising and is projected to surge by 30%, reaching 1.08 million cases annually by 2030 (GLOBOCAN) (3). HNSCC develops due to various risk factors, including smoking, alcohol consumption, betel quid chewing, poor nutrition, inadequate oral hygiene, and infections with HPV (strains 16 & 18), Epstein–Barr virus, or *Candida albicans*. While both environmental exposures and genetic mutations contribute, genetic alterations play a key role in cancer progression. Its prevalence varies by region, largely driven by tobacco and alcohol use (4, 5). Heavy users of both face a more than 35-fold increased risk (6). Some of the above-mentioned risk factors also reflect geographical, cultural, or habitual patterns (4, 5). HNSCC significantly contributes to cancer-related deaths worldwide. The challenging outlook associated with HNSCC arises from its tendency to deeply invade tissues, recur locally, and frequently spread to nearby lymph nodes (7). Although there have been advancements, the effectiveness of treatment for HNSCC in preventing tumor progression remains limited (8).

The tumor microenvironment (TME) in HNSCC is a dynamic network of cells and mediators that regulate carcinogenesis, inflammation, and immune responses. Cytokines, released by both immune and tumor cells within the TME, play a crucial role in these processes. Assessing cytokine expression provides valuable insights into tumor progression, treatment effects, and immune interactions, highlighting their significance in understanding tumor dynamics. Thus, investigating cytokines may provide deeper insights into these complex interactions (9).

Interleukin-33 (IL-33) is a cytokine belonging to the IL-1 superfamily and acts as a ligand for Suppression of tumorigenicity 2 (ST2), a member of the IL-1 receptor family (10). IL-33, a nuclear cytokine derived from tissues, is overexpressed in endothelial cells, epithelial cells, fibroblast-like cells, smooth muscle cells, and activated macrophages. IL33 primarily binds to ST2 receptor expressed in immune cells like regulatory T cells (Tregs), group 2 innate lymphoid cells (ILC2s), and mast cells *in vivo*. Additionally, it affects various other cell types, including T helper 2 (Th2) cells, T helper 1 (Th1) cells, CD8+ T cells, B cells, Natural killer (NK) cells, neutrophils, basophils, eosinophils, dendritic cells and macrophages (11, 12). IL-33 is increasingly recognized as a vital regulator of the immune system, displaying diverse effects on type-1, type-2, and regulatory immune responses (11). IL-33 is expelled from injured or necrotic cells and released into the extracellular environment. It then binds to ST2/IL-1 receptor-like 1 (a heterodimer assembled through its primary receptor), along with IL-1 receptor accessory protein (a co-receptor) resulting in nuclear signaling and immunomodulatory activity in different cells (immune, heart, tumor) (13, 14). In contrast, soluble ST2 (sST2), a form of ST2, acts as a decoy receptor, reducing IL-33's biological activity and availability (14, 15).

IL-33's pleiotropic actions make it a significant contributor to maintaining tissue balance, responding to infections, managing inflammation, and influencing cancer development (13). IL-33's action in cancer progression may involve initiating downstream signal transduction via ST2, like activating the nuclear factor-kB (NF-kB) pathway. This IL-33/ST2 axis triggers NF-kB, an important player in inflammatory and immune responses, establishing it as a significant driver of tumor growth [10]. The link between inflammatory conditions and a higher cancer risk underscores IL-33's potential involvement in cancer development [10]. IL-33, characterized as an “alarmin”, contributes to tumor-related inflammation and the advancement of tumors by modulating the immune system. Signaling molecules like IL-33, known as alarm signals, are involved in tumor associated inflammation in the TME and seem to represent a significant mechanism for tumoral immune tolerance (16).

Tumorigenesis often involves the inhibition of cell mediated (Th1) immune responses and an increase in humoral immunity (Th2). IL-33/ST2 interaction directs the differentiation of naive T cells toward a Th2 phenotype, and inhibiting this pathway enhances the anti-tumor Th1 immune response. In its early discovery, IL-33 was recognized as a catalyst for type-2 immune reactions, initiating the activity of mast cells and Th2 cells (12, 13). When extracellular IL-33 binds to ST2, it primarily triggers Th2-type immune reactions, leading to the simultaneous expression of Th2-related cytokines (10). IL-33 induction enhances Th2-type immune reactions, guiding naive T cells to synthesize IL-13 and IL-5, distinct from IL-4 (17). IL-33-induced Th2-type responses may contribute to tumor development. IL-33 is the latest identified cytokine implicated in tumor progression (18). IL-33/ST2 expression in cancer tissues is closely related to tumor progression in multiple cancer types, highlighting the potential for therapeutic targeting of the IL-33/ST2 pathway (19).

**Figure 1:**
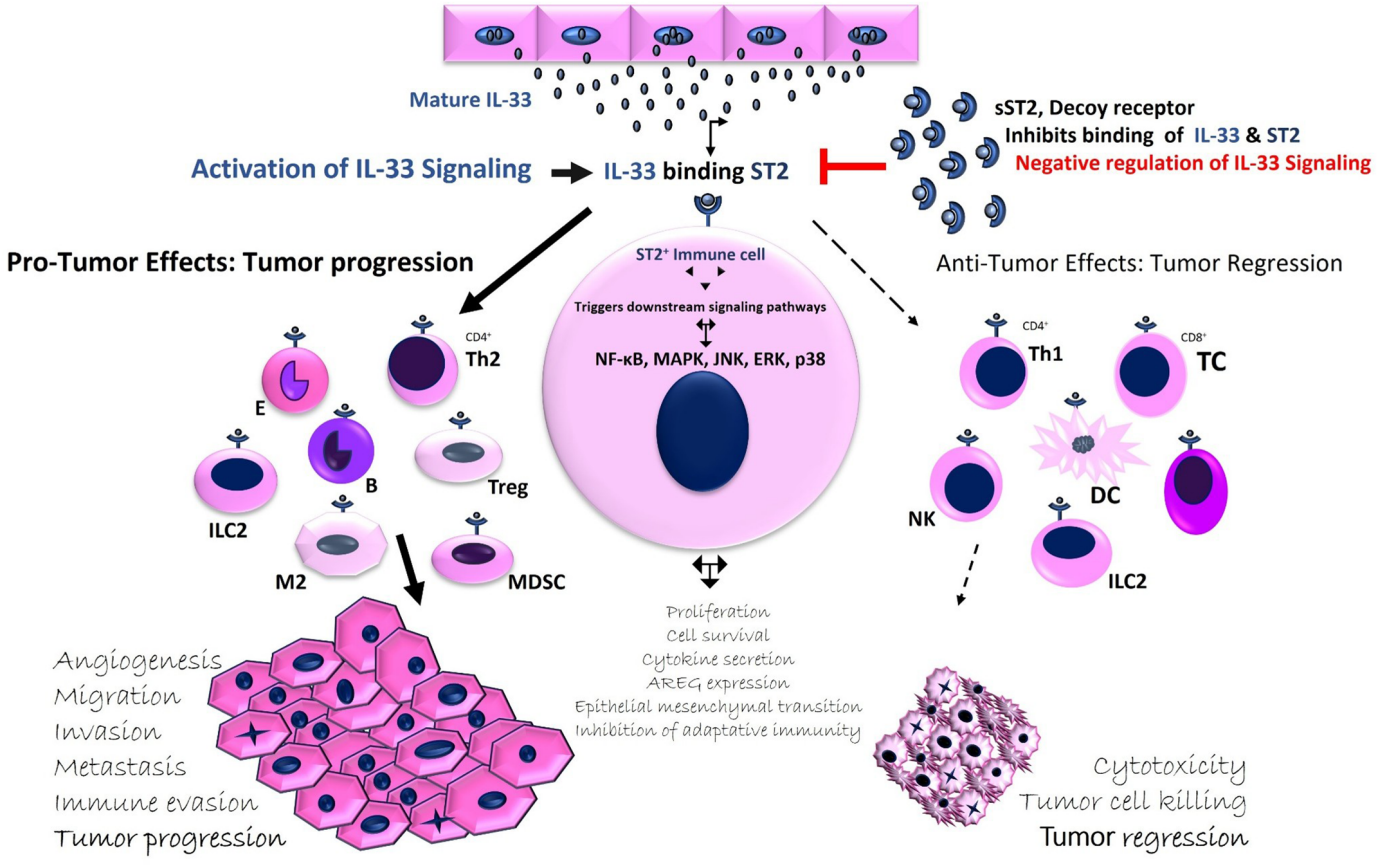
Regulation of IL-33 activity: Modulating Pro- and Anti-tumor activity in the TME. IL-33, expressed in various cell types such as endothelial, epithelial, fibroblast-like, and immune cells, is released during stress, injury, or cell damage. Binding to ST2 activates nuclear factor κB and MAPK pathways, while sST2 acts as a decoy receptor, reducing IL-33's biological activity. IL-33 plays a dual role in cancer, influencing tumor growth depending on its expression and context. It modulates the TME by affecting immune cell recruitment. IL-33 can promote tumor growth by activating immune suppressor cells (e.g., tumor-associated macrophages, Tregs, CD4+ Th2 cells) or stimulate anti-tumor immunity through innate (NK cells) and adaptive (CD4+ Th1, CD8+ T cells) responses. sST2 regulates tumor progression by sequestering IL-33.

IL-33 can exert both pro- and anti-tumor effects on immune responses. On one hand, it activates innate immunity through NK cells and adaptive immunity through CD8+ T cells and CD4+ Th1 cells (12). Recent studies also suggest that IL-33 promotes Th1 responses by stimulating Th1 cells, NK cells, iNKT cells, and CD8+ T lymphocytes. Its anti-tumor effects are linked to the activation of type 1 immune responses, including TNF-α and IFN- γ production by these immune cells (18, 20). Thus, IL-33 may play a dual role, supporting both pro- and anti-tumor responses, depending on the tumor context, expression levels, bioactivity, and the inflammatory environment (12). The interaction between IL-33 and its receptor ST2 on immune cells leads to diverse biological outcomes, which can either promote tumor progression or induce tumor regression (12) (Figure 1). Despite the growing body of evidence, IL-33's role in cancer remains a topic of debate, with both pro-tumor and anti-tumor effects observed in different contexts (13, 20–22). Nevertheless, understanding of IL-33 expression in HNSCC remains limited (10, 23, 24). In certain cancers, such as those of the ovaries, colon, and pancreas, IL-33 appears to exert an anti-tumor effect, while in others, like lung and breast cancer, it may support tumor progression (23, 25).

In such a background scenario related to different cancers, it is imperative to determine the plausible similarity of IL-33/ST2 activity specifically in HNSCC. Thus, further evaluation of evidence is necessary to substantiate the significance and clinical implications of IL-33 and ST2 in HNSCC. Hence, the objectives were to systematically review the current evidence about IL-33 and ST2's role in head and neck carcinogenesis and to assess the potential of differentially expressed IL-33 and ST2 in tumor tissues as novel biomarkers in HNSCC. Accordingly, this study aims to explore IL-33 and ST2 expression impacting the tumor dynamics, progression, and prognosis in HNSCC.

## Material and methods

### Protocol registration

This systematic review followed the guidelines stated in the Preferred Reporting Items for Systematic Reviews and Meta-Analyses (PRISMA). The study protocol was registered with the International Prospective Register of Systematic Reviews (PROSPERO) platform under the ID CRD42023447963.

### Search strategy

A comprehensive literature search was conducted using the World Wide Web as the search strategy. Relevant studies were retrieved from three major electronic databases: Web of Science, Scopus, and PubMed. The search spanned the period from January 2013 to July 2023, focusing on studies investigating the differential expression of IL-33 and ST2 in tumor tissues of HNSCC patients HNSCC.

Two researchers independently searched the electronic databases using the MeSH terms and keywords, constituting “IL-33”; “Interleukin-33”; “ST2”; “Suppression of tumorigenicity 2”; “Squamous Cell Carcinoma”; “Head and Neck Squamous Cell Carcinoma”; “Head and Neck Cancer”. As most of the relevant articles were retrieved from the Scopus database, the keywords used were as follows: (TITLE-ABS-KEY (“IL-33”) OR TITLE-ABS-KEY (“Interleukin-33”) OR TITLE-ABS- KEY (“ST2”) OR TITLE-ABS-KEY (“Suppression of Tumorigenicity 2”) AND TITLE-ABS-KEY (“Squamous cell carcinoma”) OR TITLE-ABS-KEY (“Head and Neck Squamous cell carcinoma”) OR TITLE-ABS-KEY (“Head and Neck Cancer”)) AND PUBYEAR >2012 AND PUBYEAR < 2024 AND [LIMIT-TO (LANGUAGE, “English”)] AND [LIMIT-TO (DOCTYPE, “ar”)].

### Eligibility criteria

The selected studies focused on the differential expression of IL-33 and ST2 in HNSCC tumor tissues, specifically analytical observational studies examining their role and clinical implications. Only relevant original articles published in English were included in this systematic review, and the bibliographies of the selected studies were also searched. Data on IL-33 and ST2 expression in HNSCC, obtained from The Cancer Genome Atlas (TCGA), were included. Exclusions included abstracts, editorials, short communications, book chapters, narrative reviews, systematic reviews, and meta-analyses. Studies on IL-33 and soluble ST2 (sST2) levels in body fluids of head and neck tumors were also excluded, as were studies on IL-33 and ST2 expression in tumor tissues of cutaneous squamous cell carcinoma, salivary gland tumors, and mesenchymal malignancies of the head and neck.

### Data extraction

Two researchers independently evaluated the literature sourced from electronic databases. The process began by removing duplicates, followed by screening titles, abstracts, and keywords to eliminate irrelevant articles. Full-text articles were then reviewed to determine their suitability for inclusion. The eligibility was assessed independently by two reviewers (SA, ABA), with articles included in the final selection if both reviewers agreed. In case of disagreements, a third reviewer (UH) was consulted for resolution.

Data for this systematic review were manually extracted from the selected studies. The screening of titles, abstracts, and full texts of all included articles was independently conducted by the authors (SA and ABA), with any discrepancies resolved by a third author (UH). SA collected the required data from the chosen articles for further analysis, while ABA and UH cross-checked the data for accuracy. Data were recorded on a data extraction Excel sheet. The following parameters were extracted from the selected articles: Publication details: Author(s), year of publication, and journal title; Demographic details: Population, ethnicity, and survival (if available); Clinicopathologic features: Sample site (sub-site), tumor grading and staging, lymph node metastasis (LNM); Experimental details: Study design, sample size, sample source and type, assessment method, biomolecules analyzed, source of IL-33 and ST2 expression, the association between IL-33 and ST2 expression and clinicopathologic parameters, and the relationship between IL-33 and ST2 expression and survival.

### Studies quality assessment

The methodological quality of the selected research articles was assessed using the National Institutes of Health's (NIH) “Quality Assessment Tool for Observational Cohort and Cross-Sectional Studies”, which includes 14 criteria (26). SA and ABA appraised the quality of the primary selected articles using this tool, with any disagreements resolved by UH. Each study was scored based on a system where quality was rated as follows: 0–4 (Poor), 5–10 (Fair), and 10–14 (Good), according to the total NIH score.

### Statistical analysis

The level of agreement between the two observers (SA and ABA) was measured using Cohen's kappa coefficient (κ).

## Results

### Study selection

**Figure 2:**
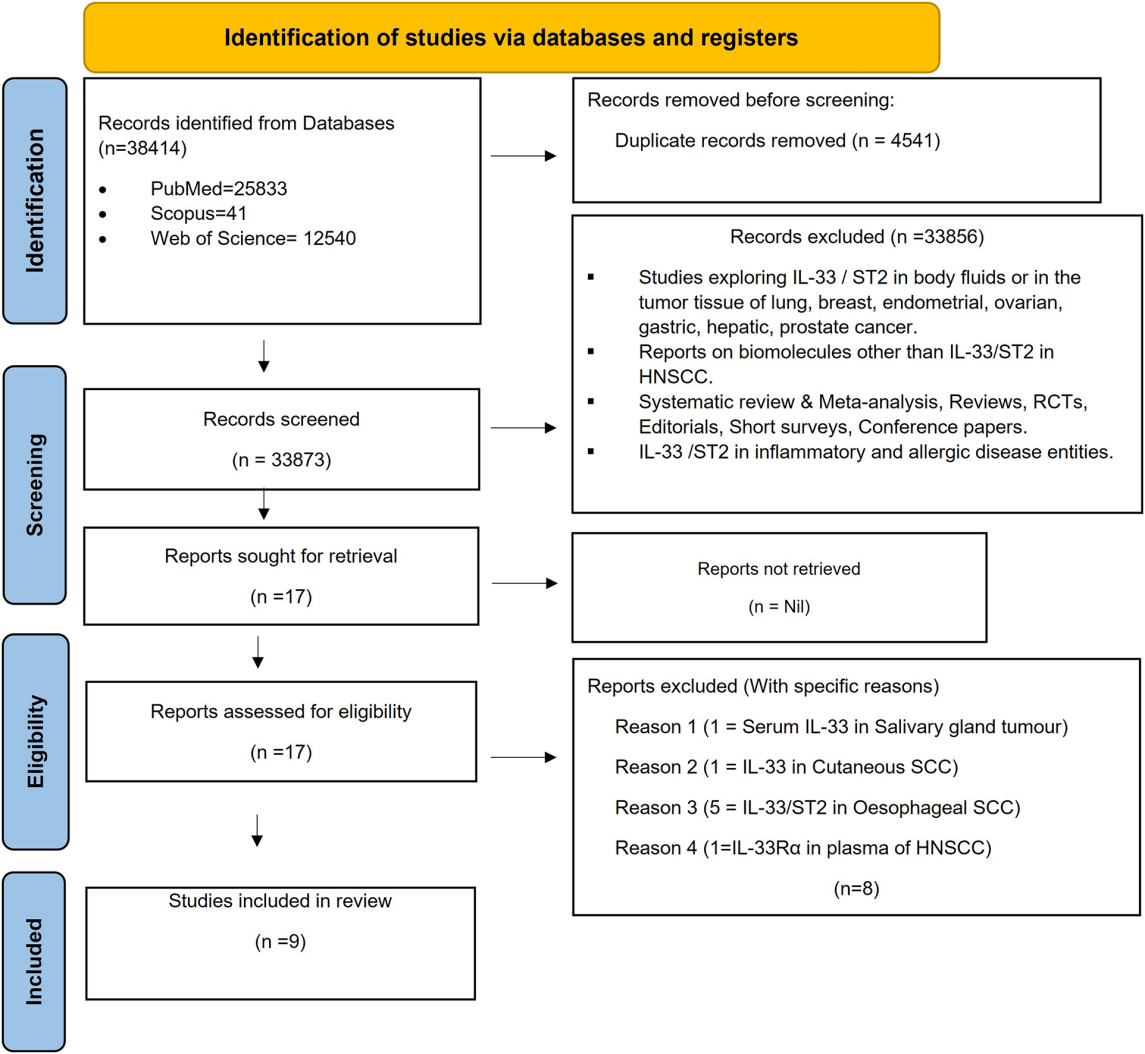
Flowchart: overview of the systematic review process following the PRISMA guidelines. A PRISMA (Preferred Reporting Items for Systematic reviews and meta-analyses) flowchart summarises the process of a systematic review, facilitating to track the number of studies included and excluded at each stage. The flowchart serves as a clear visual representation of how systematic review is conducted, highlighting methodology and rationale behind decisions at each stage.

Among the multitude of research articles, nine published studies in the English language were selected based on the eligibility criteria (7, 8, 10, 13, 16, 23, 24, 27, 28). A total of 38,414 records were first found via electronic databases (*n* = 38,414). Four thousand five hundred forty-one duplicate reports were excluded. After screening these published articles based on titles and abstracts, 33,856 were excluded and the 17 articles remaining were retrieved. Seventeen of the articles were eligible for full-text reading. Following evaluation, eight reports were excluded, which yielded nine studies for inclusion in this systematic review. The specific reasons for exclusion are mentioned in Flowchart (Figure 2) (Supplementary Table S1) Tables 1, 2 show detailed descriptions of each report. The data from the included studies, which highlighted the site (Tumor cells Stromal cells), location (Nuclear/Cytoplasmic), and percentage of HNSCC samples expressing IL-33 and ST2, showed strong reproducibility among the researchers. With a kappa (k) value of 0.90, categorized as “almost perfect agreement”, there was excellent consistency between SA and ABA, reinforcing the reliability of their evaluations.

**Table 1:** Detailed characteristics of each study enrolled in the systematic review.

Publication and demographic data			Sample data			
Reference	Population	Study design	Study cohort	Sample subsite	Source of sample	Nature of sample
Chen et al. (2013), J Pathol (7)	Taiwanese	Analytical - observation-Cross sectional	5 HNSCC 40 HNSCC (IHC)^a^	HNSCC	1. Human- (HNSCC Patient Tissues) 2. HNSCC Cell lines 3. HNSCC patients	1. HGF, CAF 2. FaDU &TW204 3. Tumor- tissue sections
Ishikawa et al. (2014), Auris Nasus Larynx (10)	Japanese	Analytical - observation-Cross sectional	81 HNSCC (IHC)^a^	OSCC-Tongue	1. HNSCC patients	1. FFPE- tissue sections
Ding et al. (2018), Carcinogenesis (24)	Chinese	Analytical -Observational – Cohort	10 paired NT & TT OSCC 5 OSCCs – (RNA sequence) 140 (Primary OSCC tissue) (IHC)^a^	OSCC-Tongue	1. Human - (OSCC Patient tissues) 2. Human OSCC cell line 3. HNSCC patients 4. HNSCC patients 5. Animal Models	1. NF, CAF 2. HSC-3 3. Fresh tumor tissue 4. FFPE sections 5. BALB/c-nu/nu T cell-deficient mice
Wen et al. (2019), Cancer Immunol Immunother (16)	Chinese	Analytical - Observational- Case control	68 HNSCC (IHC)^a^ 10 pairs LSCC- TT & A-NT 15 LSCC-PBMCs 20 HC -PBMCs	LSCC	1. HNSCC patients 2. HNSCC patients 3. Human LSCC cell lines 4. HNSCC patients 5. Healthy controls	1. FFPE – tissue sections 2. Tumor tissue & A-NT 3. SNU899- TSNs 4. Peripheral blood 5. Peripheral blood
Lin et al. (2021), Cancers (Basel) (8)	Taiwanese	Analytical -Observational- Case control	40 HNSCC (IHC)^a^	HNSCC	1. Human- (HNSCC Patient Tissue) 2. HNSCC Cell lines 3. HNSCC patients 4. Animal Models	1. HGF, CAF 2. FaDu & TW204 3. Tumor tissue 4. BALB/c nude mice
Peng et al. (2021), Front Oncol (13)	Chinese	Retrospective- Observational-Cross-sectional	520 HNSCC (IHC)^a^	LSCC OPSCC OCSCC	Human (cBioPortal for Cancer Genomics)	1. Tumor tissue
Adamski et al. (2021), Biomedicines (23)	Polish people	Analytical- Observational-Cross-sectional	95- Chemonaive OSCC (IHC)^a^	OSCC	1. HNSCC patients	1. FFPE -TMA
Zhao et al. (2022), Pathol Res Pract (27)	Chinese	Analytical- Observational-Cross-sectional	94 LSCC (IHC)^a^ 20 LSCC (FC)	LSCC	1. HNSCC patients 2. HNSCC patients	1. FFPE tissue sections 2. Fresh Tumor tissue/A-NT
Zhao et al. (2023), Br J Cancer (28)	Chinese	Analytical -Observational- Case control	30 OSCC tissues (IHC)^a^ 45 OSCC tissues (RNA)	OSCC	1. HNSCC Patients 2. TCGA database 3. HNSCC Cell lines 4. Animal Models	1. FFPE tissue sections 2. Activated T cells *in situ* & Peripheral blood 3. HN6, Cal27, Hsc3 and SCC7 cells 4. NCG and C57BL/6mice

A-NT, adjacent normal tissue; CAF, carcinoma associated fibroblasts; FC, flow cytometry; FFPE, formalin fixed paraffin embedded; HGF, high gingival fibroblasts; IHC, immunohistochemistry; LSCC, laryngeal squamous cell carcinoma; OSCC, oral squamous cell carcinoma; OPSCC, oropharyngeal squamous cell carcinoma; OCSCC, oral cavity squamous cell carcinoma; PBMNc, peripheral blood mononuclear cells; TMA, tissue micro array; TT, tumor tissues; TSNs, tumor culture supernatants.

^a^ Clinicopathologic characteristics.

**Table 2:** Summary of study characteristics, assays, IL-33/ST2 sources, and key findings.

Authors	Markers analyzed	Assays performed	Source of IL-33 & ST2 expression	Results
Chen et al. (7)	IL-33	IHC	Tumor cells CAFs	IL-33 expression TCs; Positive staining in 90% of samples CAFs; Positive staining in 52% of samples ➢ High expression level of IL-33 in CAFs correlated with increased expression level of IL-33 in TCs
	∝ SMA & Vimentin EMT markers & Regulators	WB & IHC Trans well Migration Assay Invasion Assay WB & ELISA RNA extraction & qRT-PCR	HGFs CAFs HNSCC Cell lines	➢ Increased expression of ∝-SMA & Vimentin in CAFs than HGFs. ➢ Increased migration ability and invasiveness in HNSCC cell line treated with CAFs. ➢ IL-33 is significantly over-expressed in CAFs ➢ IL-33 mRNA upregulation in CAFs. ➢ IL-33 induces the EMT process in HNSCC cell lines and activates the expression of certain EMT-related genes.
Ishikawa et al. (10)	IL-33 ST2	IHC	Tumor cells	IL-33 expression; 100% samples TC: Nucleus & Cytoplasm MES; TC:41.59 ± 23.89% +ve ST2 expression; 95% samples TC: Cytoplasm & Cell membrane MES; TC:22.14 ± 19.2% +ve ➢ IL-33 expression showed a significant positive correlation with ST2 expression.
	Anti-tryptase antibody CD34	Mast cell density (MCD) Microvessel density (MVD)		➢ Mean MCD was 12.54 ± 11.60%. ➢ IL-33 expression significantly correlated with MCD. ➢ MVD was significantly higher in the high IL-33 group than in the low IL-33 group
Ding et al. (24)	IL-33 Ki-67 CD31	IHC	Tumor cells Stromal cells NF CAFs	➢ IL-33 was mainly expressed in the tumor stroma, but rarely in the tumor nest, which indicated that IL-33 is indispensable for the stromal microenvironment. ➢ Lnc-CAF fosters the CAF phenotype via IL-33, aiding TC proliferation. ➢ Lnc-CAF/IL-33 expression increases during NF-to-CAF transformation.
	Lnc-CAF *α*-SMA FSP-1 IL-33 Ki-67	Isolation of CAF RNA-seq -Novel Bioinformatics Co Immunofluorescence (IF) Fluorescent *in situ* hybridization (FISH) RNA isolation & qRT-PCR Lnc-CAF- gene expression analysis Trans well migration assay Cell treatment & transfection Immunoblotting Flowcytometry (FC) RNA immunoprecipitation assay Exosome isolation/characterization Tumor mouse model	NF CAFs HNSCC Cell lines	➢ Lnc-CAF/IL-33 signaling in stromal fibroblasts enhances HSC3 cell growth. ➢ Lnc-CAF stabilizes IL-33 by preventing its degradation through the p62-dependent autophagy–lysosome pathway, supporting tumor progression. ➢ Tumor-derived exosomal Lnc-CAF amplifies its levels in stromal fibroblasts, establishing a positive feedback loop. ➢ High Lnc-CAF/IL-33 expression is associated with advanced OSCC stages. ➢ Lnc-CAF knockdown suppresses OSCC tumor growth.
Wen et al. (16)	IL-33 Foxp3	IHC	Tumor cells Stromal cells (CAFs, MDSCs) Treg cells	➢ Stromal IL-33 expression was remarkably upregulated in advanced stage Vs early-stage tumors. ➢ Stromal IL-33 expression positively correlated with Foxp3^+^ Treg infiltration as well as poor prognosis. ➢ IL-33 stimulation led to an increased percentage of Tregs. ➢ IL-33 boosted Treg numbers and prompted the production of immune-dampening cytokines.
	GATA3 CD83 CD80 CD86 CD40 CD3 CD4 ST2 IL-10 TGF-β1	T cell subpopulation isolation Cell culture cell suppression assays Cell proliferation assay Preparation of DC ELISA FC Cytokine detection	T cell subpopulation HNSCC cell lines	➢ ST2 expression in Tregs from HNSCC tissue was higher than in Tregs from peripheral blood. ➢ IL-33 promotes Treg expansion and stimulates the production of suppressive cytokines.
Lin et al. (8)	IL-33 CXCR4	IHC	Tumor cells	IL-33 expression TCs Negative in 55% samples Positive in 45% samples CXCR4 TCs Negative in 32.5% samples Positive in 67.5% samples ➢ Strong correlation observed between IL-33 expression and CXCR4 expression.
	ST2 SDF1 IL-6 IL-8 MMP-2 MMP-9	Cell culture WB analysis RNA extraction qRT PCR Migration & Invasion assay FC Organotypic 3D Culture ELISA & Sphere culture Assay for chemo & Radiosensitivity *In vivo* assay	HGF, CAFs HNSCC cell lines	➢ CAF-induced IL-33 reciprocally enhanced cancer cell autocrine IL-33 production and CXCR4 upregulation, activating SDF1/CXCR4 signaling and promoting cancer progression. ➢ Higher expression of ST2 in the cloned IL-33 overexpressing HNSCC cells than in the control HNSCC cells.
Peng et al. (13)	IL-33	mRNA data seq TCGA	Tumor Tissue Sources of IL-33 Epithelial cells Endothelial cells Pericytes Fibroblasts SMC	mRNA expression level of IL-33 ➢ High IL-33 expressing tumors accumulated more types of immune cells (T_reg_ cells, CD8^+^ T cells, macrophages, M1 macrophages, monocytes, DCs, neutrophils, eosinophils, mast cell, naive CD4^+^ T cells, CD4 ^+^ T cells)
	CTLA-4 PD-1 PD-L1 IFN-γ CYT	Differential gene expression analysis cBioPortal platform		➢ Molecular markers were all higher in the high IL-33 group than in the low IL-33 group.
Adamski et al. (23)	IL-33 PD-L1	IHC	Tumor cells TILs	IL-33 expression TCs ➢ 15.79% samples showed IL-33 expression in >1% of TCs nuclei. TILs ➢ Positive expression of IL-33 in TILs was observed in 18.94% samples. PD-L1 TCs ➢ 46.31% samples showed positive expression of PD-L1 in >10% of TCs. TILs ➢ 31.63% samples showed PD-L1 positivity in >20% of TILs. No correlation was found between PD-L1 and IL-33 expression.
Zhao et al. (27)	IL-33 FOXP3 ∝ SMA	IHC/IF	Tumor cells Stromal cells Immune cells Fibroblasts CAFs	➢ In HNSCC, there is a notable increase in Tregs. ➢ Higher frequency of ST2-expressing Tregs in the IL-33⁺ microenvironment. ➢ IL-33 remarkably expressed on TCs, ICs and CAFs, with the latter representing the primary source.
	ST2 CD45 CD3 CD4 Ki-67 PD1 CD25 CTLA4 CD31 EpCAM PDGFR∝	Cell separation FC IF		➢ Increase in IL-33 within the stroma stems from CAFs and correlates with the existence of ST2^+^ Treg cells. ➢ The number of IL-33-positive CAFs correlated with ST2 expression on Tregs. ➢ Increased ST2^+^ Tregs in the IL-33^+ve^ TME Vs IL-33^−ve^ ➢ Increased Ki-67 and CTLA-4 in the ST2⁺ Tregs Vs ST2⁻ Tregs
Zhao et al. (28)	IL-33 ST2	IHC	Tumor cells NFs CAFs Immune cells HNSCC cell lines	IL-33 & ST2 expression ➢ Expression of IL-33 and ST2 absent in normal epithelium and upregulated during carcinogenesis. ➢ ST2 was mainly expressed in TCs. ➢ IL-33 showed more stromal expression in CAFs and vascular endothelial cells. ➢ The expression of ST2 in tumor tissues was significantly higher than that in normal tissues. ➢ IL-33 was also up-regulated in tumor tissues of OSCC and further enriched in metastatic niche
	pSTAT3 p-JAK PD-L1 JAK2 STAT3 NF-κB p-NF-κB IFNGR1 CD8 Foxp3	RNA analysis & qPCR Isolation and primary culture of fibroblasts Cell Culture RNA analysis T cell-mediated tumor cell killing assay Cell transfection IF WB FC ELISA Experimental animals		➢ High IL-33/ST2 levels are associated with reduced infiltration of activated T cells *in situ* and in peripheral blood ➢ IL-33/ST2 signaling enhances PD-L1 expression in OSCC ➢ ST2-high tumor cells suppress the tumor-killing function of human CD8+ T cells via PD-L1. ➢ ­ ST2 knockdown combined with anti-PD-L1 therapy exhibits enhanced anti-tumor effects in OSCC.

CAFs, carcinoma associated fibroblasts; CD, cluster differentiation, CTLA-4, cytotoxic t-lymphocyte antigen; CTY- immune cytolytic activity; DC, dendritic cells; ELISA, enzyme linked immunosorbent assay; FC, flow cytometry, HGF, high gingival fibroblast; INF, interferon; IC, immune cells; IF, immunofluorescence; IHC, immunohistochemistry; IFN-γ, interferon gamma; Lnc-CAF, long non-coding RNA associated with CAF; MDSCs, myeloid derived stem cells; MES, mean expression scores; NF, normal fibroblasts; PD-L1, programmed death Ligand 1; qRT-PCR, quantitative real-time reverse transcription polymerase chain reaction; SDF1/CXCR4, stromal-derived factor 1/C-X-C motif chemokine receptor 4; SMC, smooth muscle cells; TCGA, The cancer genome atlas, TILs, tumor infiltrating lymphocytes; Treg, regulatory T cells; TC, tumor cells; TME, tumor microenvironment; WB, Western blot.

### Study quality assessment

**Figure 3:**
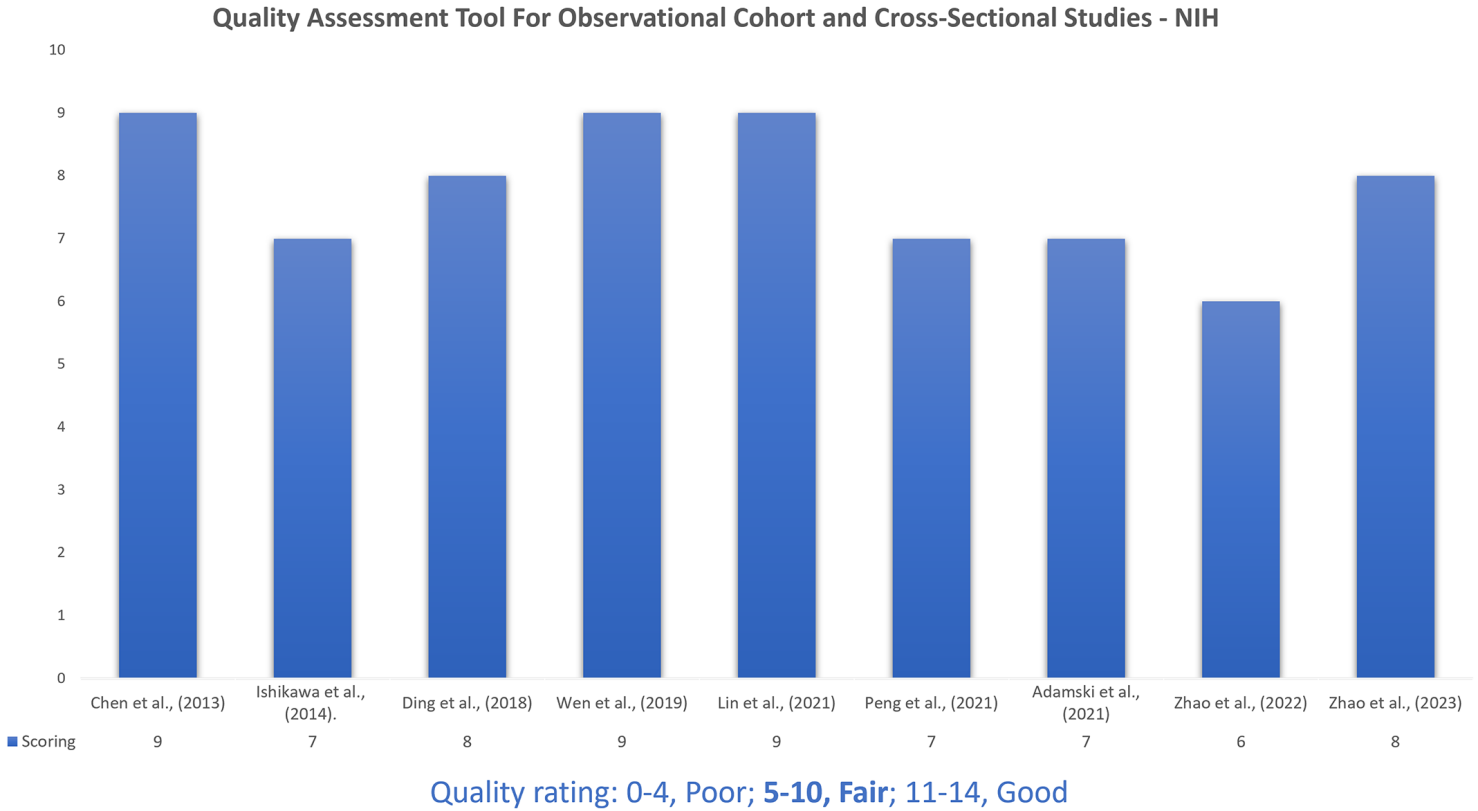
The National Institutes of Health's (NIH) quality assessment tool for observational cohort and cross-sectional studies. The quality assessment tools from the NIH for cohort and cross-sectional observational studies are designed to help analyse the risk of bias and methodological rigor in original research work. These aids provide a structured approach for evaluating various aspects of study design, including sample size, data collection methods, outcome measures and statistical analysis performed.

After assessment of the studies, the NIH scoring ranged from 6 to 9. The quality of the nine articles included was a fair rating score ranging between 5 and 10 (Figure 3).

### Study characteristics

Nine studies fulfilling the inclusion criteria were analyzed (7, 8, 10, 13, 16, 23, 24, 27, 28). These studies were primarily observational and analytical by nature and were predominantly carried out in the Southeast Asian population (7, 8, 10, 13, 16, 24, 27, 28). The publication details, demographics, and sample data are tabulated. Table 1 provides detailed characteristics of each study included in the systematic review. The objectives of the included studies aligned with the review question.

IL-33 was evaluated in all nine studies. Of these, tumor tissues of HNSCC patients expressing IL-33 were assessed using immunohistochemistry (IHC) in eight original studies (7, 8, 10, 16, 23, 24, 27, 28), while one study employed mRNA sequencing (TCGA) (13) (Table 2). IL-33 expression in tumor cells, detected by IHC staining, was analyzed in eight studies (7, 8, 10, 16, 23, 24, 27, 28) whereas stromal cell expression was evaluated in six studies (7, 16, 23, 24, 27, 28).

High gingival fibroblasts (HGF) and carcinoma associated fibroblasts (CAF) extracted from HNSCC patient samples were used to demonstrate IL-33 expression in *in vitro* assays (7, 8, 16, 24, 27, 28). Additionally, HNSCC cell lines, with CAFs, were used for both *in vitro* and *in vivo* experiments (7, 8, 16, 24, 28).

Beyond IHC analysis of tumor tissues, supplementary techniques like flow cytometric (FC) analysis, enzyme linked immunosorbent assay (ELISA), Western blot (WB) analysis, RNA isolation, and quantitative real-time reverse transcription polymerase chain reaction (qRT-PCR) were employed to assess IL-33 protein or gene expression in cell culture supernatants, cell lysate, and harvested cells (7, 8, 16, 24, 27, 28).

ST2 expression was evaluated in five studies (8, 10, 16, 27, 28). Among these, two studies (10, 28) assessed ST2 expression in tumor tissues using IHC, specifically evaluating its presence in tumor cells. Surface or intracellular ST2 expression in individual cells from TME or cultured cells was analyzed using FC and immunofluorescence (IF), whereas WB was used to detect and quantify ST2 expression in tissue extracts, as well as harvested or cultured cells (8, 16, 27).

*In vivo* assays using animal models were conducted in three studies (8, 24, 28).

All the studies included in the review examined the association between IL-33 and ST2 expression in HNSCC and various clinicopathological characteristics (7, 8, 10, 13, 16, 23, 24, 27, 28) (Table 3)_._

**Table 3:** Relationship between IL-33 and ST2 expression, clinicopathologic features, and prognosis.

Authors	IL-33/ST2 expression & Clinicopathologic features	IL-33/ST2 expression & Prognosis
Chen et al. (7)	• In high IPGS, there is an observed upregulation of IL-33 in both CAFs & TCs. • Significant relationship between expression of IL-33 in CAFs & nodal stage.	• Elevated levels of IL-33 in TCs and CAFs were associated with reduced nodal metastasis-free survival. • Increased expression IL-33 is linked with poor prognosis regarding nodal metastasis-free survival.
Ishikawa et al. (10)	• Expression of IL-33 in TCs was significantly high in HNSCC patients with LR & NR. • There was a notable rise in ST2 expression within the TCs among patients experiencing NR.	• Elevated levels of IL-33 indicate a risk factor for a poorer prognosis. • A significantly worse prognosis was associated with high expression levels of IL-33. • The expression of ST2 was linked to a poorer prognosis.
Ding et al. (24)	• Elevated expression of Lnc-CAF and IL-33 correlated with advanced tumor stage. • Patients with higher expression levels of Lnc-CAF and IL-33 showed increased proliferation index, as indicated by higher Ki-67 levels.	• A high level of Lnc-CAF, rather than IL-33, was predictive of a poor clinical survival outcome for patients with OSCC. • A strong expression of Lnc-CAF was indicative of a poor prognosis.
Wen et al. (16)	• Tumors in advanced clinical stages displayed a notably higher count of stromal IL-33^+^ cells.	• The presence of stromal IL-33^+^ cells is linked to a poorer prognosis. • Patients exhibiting high expression levels of IL-33 tend to have poorer survival outcomes. • Patients with a higher count of stromal IL-33^+^ cells experienced poorer OS and PFS compared to those with fewer IL-33^+^ cells.
Lin et al. (8)	• The IL-33 and CXCR4 expression, along with their co-expression, displayed a notable correlation with tumor differentiation, LNM, and TNM stage.	• IHC confirmed a significant correlation between IL-33 and CXCR4 expression with DFS. Additionally, the co-expression of IL-33 and CXCR4 indicated a poorer outcome. • Patients negative for both IL-33 and CXCR4 expression showed a longer DFS compared to those who were negative for IL-33 but positive for CXCR4. • The simultaneous expression of IL-33 and CXCR4 in TCs displayed a significant correlation with shorter DFS, suggesting a poorer outcome.
Peng et al. (13)	• The higher the expression level of IL-33, the earlier the pathologic T stage of the tumor.	• A high expression of IL-33 in tumor samples is often associated with an unfavourable prognosis in LSCC. • A high expression level of IL-33 in LSCC often correlated with poorer OS rates. • In OPSCC, the expression level of IL-33 did not hold any predictive value for prognosis. • In OCSCC, a high expression of IL-33 in tumor samples appeared to be linked with a more favourable OS outcome.
Adamski et al. (23)	• Tumors in more advanced stages showed a tendency to express higher levels of IL-33 compared to those in early stages.	• In OSCC, the expression of IL-33 was infrequent and did not demonstrate a significant impact on patients’ survival, indicating no prognostic significance. • IL-33 expression was seldom observed and did not show any notable influence on patients’ survival.
Zhao et al. (27)	• The presence of IL-33^+^ CAFs showed a significant association with both LNM and advanced clinical stages. • The abundance of ST2^+^ Tregs was significantly linked to advanced clinical stages.	• The combination of high ST2 expression on Tregs and the presence of IL-33-expressing CAFs was linked to poorer survival outcomes. • The elevated ST2 expression on Tregs, along with the concurrent high ST2 on Tregs and the presence of IL-33-positive CAFs, predicts a poor prognosis in laryngeal cancer.
Zhao et al. (28)	• IL-33 & ST2 up-regulated in tumor tissues of OSCC Vs Normal tissues. • IL-33 is further enriched in metastatic niche.	• High ST2 had short overall survival time in patients with OSCC. • High IL-33 had worse OS than those with low IL-33 expression in patients with OSCC.

CAFs, carcinoma associated fibroblasts; CXCR4, C-X-C chemokine receptor 4; DFS, disease free survival; IPGS, invasive pattern grading system; IL-33, interleukin-33; IHC, immunohistochemistry; LNM, lymph node metastasis; LSCC, laryngeal squamous cell carcinoma; Lnc-CAF, long non coding RNA associated with CAF; LR, local recurrence; NR, nodal recurrence; OCSCC, oral cavity squamous cell carcinoma; OPSCC, oropharyngeal squamous cell carcinoma; OS, overall survival; PFS, progress-free survival; ST2, Suppression of tumorigenicity 2; TCs, tumor cells, Treg, regulatory T cells.

### Study outcome

IL-33 expression has been observed in tumor cells, and stromal cells, including CAF, endothelial cells, tumor infiltrating lymphocytes (TIL), and other immune cells within the TME of HNSCC. IL-33 expression in tumor cells was analyzed in eight studies, with its localization detected in both the nuclei (chromatin-associated cytokine) and the cytoplasm. Some investigators reported sparse IL-33 expression within the tumor nest but high expression in the surrounding stroma (24). IL-33 expression in stromal cells was analyzed in six studies, consistently showing that IL-33 is predominantly expressed in CAFs. Additionally, IL-mRNA was found to be upregulated in CAFs (Table 2).

Furthermore, WB analysis demonstrated IL-33 overexpression in CAF lysates compared to HGFs, while ELISA confirmed elevated IL-33 levels in CAF-conditioned medium (CAF-CM). Microarray and qRT-PCR further validated increased IL-33 mRNA expression in CAFs. The Transwell migration assay showed a 2- to 3-fold increase in HNSCC cell migration when exposed to CAF-CM compared to the control. CAF-CM also enhanced invasiveness in HNSCC cell lines as demonstrated by invasion assay. WB analysis confirmed EMT induction in HNSCC cells co-cultured with CAF-CM, indicated by a shift from epithelial to mesenchymal markers and upregulation of EMT regulators. Similarly, recombinant IL-33 treatment induced EMT marker changes and morphological transition, reinforcing IL-33's role in EMT induction in HNSCC cells. *In vivo* assays using animal models demonstrated that IL-33-overexpressing HNSCC cells exhibit greater tumorigenicity compared with control cells.

The collective findings highlight the critical role of IL-33 in CAF-induced cancer aggressiveness, primarily due to its overexpression in CAFs. Elevated IL-33 levels in CAFs strongly correlate with IL-33 expression in tumor cells (7). Several studies have reported a positive association between IL-33 expression in CAFs and tumor cells. Furthermore, significant associations have been noted between IL-33 expression in CAFs and key clinicopathological features, including advanced TNM staging and LNM. Higher IL-33 levels in CAFs have been linked to poor clinical outcomes, particularly reduced nodal metastasis-free survival (Table 3). Overall, the majority of the studies suggest that IL-33 mainly functions as a pro-tumorigenic factor in HNSCC.

In HNSCC, both tumor cells and Tregs express ST2, with its expression being most prominent in the cytoplasm and membrane of tumor cells. A significant increase in Tregs has been observed in HNSCC, with a higher frequency of ST2-expressing Tregs in IL-33-enriched TME. Elevated IL-33 levels in the stroma, primarily derived from CAFs, correlate with the presence of ST2^+^ Tregs. Additionally, the number of IL-33-positive CAFs has been positively associated with ST2 expression on Tregs.

*In vitro* experiments, including cell suppression assays, FC, and ELISA, have demonstrated that IL-33 promotes Treg expansion and induces the production of suppressive cytokines like IL-10 and TGFβ (16). Notably, high IL-33/ST2 levels are linked to reduced infiltration of activated T cells within the TME, suggesting an immunosuppressive role. IL-33-treated HNSCC cells formed 3D tumor organoids, where IL-33/ST2 activation induced p-STAT3 nuclear localization and PD-L1 upregulation, promoting immune evasion. *In vivo* studies showed that ST2-high tumors inhibit CD8+ T cell tumor-killing via PD-L1, while ST2 knockdown combined with anti-PD-L1 therapy enhances anti-tumor effects in HNSCC (28).

Collectively, high ST2 expression in tumor tissues shows a significant correlation with IL-33 expression (10). A higher abundance of ST2^+^ Tregs is strongly associated with advanced clinical stages (27). Elevated ST2 expression in tumor cells is particularly notable in patients with nodal recurrence (10). Moreover, ST2 expression on Tregs is closely associated with the presence of IL-33-positive CAFs. The combined presence of high ST2 expression on Tregs and IL-33-expressing CAFs correlates with poorer survival outcomes in HNSCC. In HNSCC patients, high ST2 expression is linked to shorter overall survival (16, 27, 28) (Table 3).

## Discussion

The review findings indicate that several studies have reported elevated IL-33 expression in HNSCC tumor tissues compared to adjacent normal tissues (7, 8, 10, 13, 16, 24, 27, 28). Likewise, increased IL-33 expression has been observed in ESCC tumor tissues (29–31). Ishikawa et al., found that IL-33 expression in HNSCC increased with the tumor progression and recurrence (10) Elevated IL-33 expression is associated with a more aggressive disease course and a worse prognosis in HNSCC patients (8, 10, 16, 28). Similarly, Yue et al., reported IL-33 overexpression in ESCC, linking it to increased metastasis and invasion (30). However, Li et al., observed higher IL-33mRNA and protein IL-33 levels in normal tissues than in paired HNSCC tumors. Lower IL-33 levels were associated with advanced T stages, LNM, and later clinical stages (32). Similarly, Yang et al., noted downregulated IL-33 and ST2 in lung squamous cell carcinoma and adenocarcinoma, with IL-33 inversely correlating with tumor grade, size, and progression, supporting a role in tumor immune surveillance (33). Furthermore, evidence suggests that the expression of IL-33 in tumor cells may enhance immunogenicity and foster type 1 antitumor immune responses through NK and CD8+ T cells (34). It has been proposed that IL-33's role in HNSCC shifts depending on its cellular source. Specifically, when IL-33 originates from stromal cells, it fuels tumor growth, but when it comes from epithelial cells, it hampers it (13). Limited evidence exists on IL-33 and ST2 influencing the development and progression of HNSCCs. This review may be notable as the first exploration emphasizing IL-33/ST2 expression in HNSCC tissues, suggesting a potential role in tumor dynamics.

In addition to its expression in tumor cells, IL-33 is also highly expressed in the TME, particularly within stromal cells such as CAFs, where it plays a crucial role in tumor progression and immune modulation (7, 16, 24, 27). The TME is a complex ecosystem that significantly contributes to the development of HNSCC (35). It consists of stromal cells like fibroblasts, inflammatory cells, endothelial cells, and the extracellular matrix, all of which influence tumor initiation and progression (7). Among these, CAFs are the predominant non-immune cell type in the TME, playing a key role in tumor-stromal interactions (24). CAFs are recognized for their substantial contribution in providing specific growth factors or cytokines, which facilitate tumor interaction and contribute to the progression of cancer (7, 9). Several investigators noted that IL-33, predominantly present in the stromal environment, exhibits high expression within CAFs, contributing significantly to the tumor invasiveness driven by CAFs (7, 16, 24, 27). Upregulated IL-33 expression spurred the activation of CAFs, contributing to a phenotype that facilitated CAF-supported tumor growth in HNSCC (24).

CAFs escalate cancer invasiveness by modulating IL-33 signaling in the TME through paracrine and autocrine influences, where paracrine signaling enhances tumor aggressiveness via IL-33/ST2 and stromal-derived factor 1/C-X-C motif chemokine receptor 4 (SDF1/CXCR4) pathways, while autocrine signaling sustains cancer cell self-production of IL-33, further amplifying CXCR4 expression and tumor progression (8). These results align with earlier studies in HNSCC, demonstrating that CAFs express IL-33 abundantly, contributing to the induction of tumor cell invasiveness through EMT in cancer cells (24). IL-33 also fosters the properties of stemness through crosstalk involving CAFs and tumor cells (8). IL-33 has the potential to regulate the tumor immune microenvironment in HNSCC (13). The overexpression of IL-33 in CAFs fundamentally reprogrammes the interaction between HNSCC tumor cells and CAFs, ultimately propelling tumor progression (7). Even the IL-33 mRNA levels markedly increased in CAFs, alongside elevated long non-coding RNAs associated with CAF (Lnc-CAF), stabilizing IL-33 by inhibiting its degradation through the autophagy-lysosome pathway (24).

IL-33 in the tumor stroma fosters immune tolerance and suppression through Tregs and MDSCs (16, 36). Increased IL-33 expression in CAFs sustains or triggers the activation of immune-suppressive cells like tumor-associated CD4+ Th2 cells, Tregs, and macrophages, thereby promoting tumor progression and metastasis (25). IL-33 is essential in mediating immunosuppression by Tregs in cancers, forming the basis for the interaction between Tregs and the TME. The accumulation of Tregs and inhibition of the proliferation of T effector cells can be regulated by CAFs (27). Given that CAFs are a significant source of IL-33 and contribute to immune evasion, it is suggested that stromal IL-33 could potentially exploit opportunities to activate ST2-positive tumor cells, thereby inducing programmed cell death ligand-1 (PD-L1) expression and promoting immunosuppression (28, 37). Cancer and immune cells interact through PD-L1 and its receptor, programmed cell death protein 1 (PD-1). PD-L1 expression on cancer cells enables immune evasion by activating the PD-1 checkpoint on cytotoxic CD8^+^ T lymphocytes, suppressing their activity (23).

ST2 signaling promotes PD-L1 expression via the JAK-STAT3 pathway and enhances IFN- γR, amplifying IFN- γ-driven PD-L1 upregulation. Tumors with high ST2 expression exhibit increased IL-33 and IFN- γ-mediated PD-L1, reducing activated T cells in the TME and driving tumor progression. However, such tumors may respond better to anti-PD-1/L1 therapy (28). Additionally, IL-33/ST2 signaling enhances Treg activity (e.g., CTLA-4, PD-1) and expands Tregs, further suppressing T cell proliferation. Inhibiting the IL-33/ST2 axis alongside immune checkpoints may restore T cell function and improve antitumor immunity (38, 39).

In addition, tumor-derived IL-33 drives Treg expansion and enhances their immunosuppressive functions across multiple cancer types. ST2^+^ Tregs are highly responsive to IL-33, and their abundance correlates with the density of IL-33-expressing CAFs. Functional analyses show that IL-33 promotes the expansion of Foxp3^+^GATA3^+^ Tregs, reinforcing their suppressive activity by increasing IL-10 and TGF-β1 production while inhibiting responder T cell proliferation. In HNSCC, IL-33 is linked to increased ST2^+^ Tregs, suggesting IL-33/ST2 signaling regulates Tregs within the TME. Stromal IL-33 is associated with poor prognosis, likely due to its role in enhancing Treg-mediated immune suppression. Targeting stromal IL-33 or modulating Tregs presents a promising strategy to counteract immunosuppression and improve HNSCC immunotherapy outcomes (16).

This review emphasizes the relation between increased ST2 expression on Tregs and IL-33 signaling originating from CAFs in HNSCC. This accentuates the unique contributions of CAFs and Tregs to mold the immune-evading environment of tumors through IL-33/ST2 interactions (27). IL-33 positive CAFs in the TME potentially stimulate the expansion of ST2-positive Tregs, reinforcing their function. High levels of ST2-positive Tregs are clinically linked to reduced disease-free survival, affirming their suppressive nature and confirming their role in immune evasion. Elevated levels of ST2-positive Tregs alongside IL-33 in CAFs correlate with decreased survival, indicating a potential synergistic action among ST2-positive Tregs and IL-33 positive CAFs in promoting tumor development (27). The IL-33/ST2 axis amplifies the malignancy traits of tumors by impacting both the tumor and its surrounding microenvironment (10). The findings unveiled a multifaceted tumor-promoting environment influenced by the IL-33/ST2 axis in HNSCC (28). Targeting IL-33 could serve as a therapeutic strategy to enhance prognosis and survival in HNSCC patients (7).

One of the key strengths of this analysis is that it represents the first comprehensive attempt to evaluate the role of IL-33 and ST2 in the development of HNSCC. While the role of IL-33 and ST2 in solid tumors has been well established, their specific involvement in HNSCC has remained underexplored. This study systematically examined the literature for their expression in tumor tissues, emphasizing IHC evaluation, complemented by mRNA expression analysis and *in vitro*/*in vivo* experiments using HNSCC cell line, HGFs & CAFs. IHC provides direct visualization of IL-33 and ST2 protein expression, assessing the spatial distribution, localization, and intensity within tumor compartments, including the TME and stroma. Unlike mRNA analysis, which reflects gene expression at the transcriptional level. By integrating IHC with other complementary techniques, this study enhances the reliability of the findings and laying a foundation for future research on IL-33 and ST2 as potential biomarkers or therapeutic targets in HNSCC.

However, the role of serum IL-33 and ST2 in the evolution of HNSCC remains to be explored. Aarstad HH et al. found ST2 to be a potential predictor of survival, suggesting its prognostic value as a cancer marker (40). However, a previous study by the same group reported no association between plasma IL-33R*α*/ST2 and prognosis (41). The role of serum IL-33 in HNSCC remains unexplored. Serum IL-33 shows promise as a diagnostic and prognostic marker in NSCLC (42) and is elevated in various cancers, including hepatic, prostate, gastric, endometrial, and breast cancer (43–48). Serum IL-33 and ST2 may serve as non-invasive diagnostic markers for breast cancer (48). ST2 receptor activation by IL-33 provides a stimulus for the growth and metastasis of several types of cancers (15, 49) and inhibits anti-tumor immunity (50). In the early prediction of cancer, it has been advocated that IL-33 and ST2 may serve as potential biomarkers (51).

This literature review evaluating the prognostic significance of IL-33 and ST2 expression in HNSCC tumor tissues has several limitations. First, the analysis was restricted to tumor tissue expression, as there are hardly any reports on IL-33 and ST2 expression in the body fluids of HNSCC, which may have provided additional insights into their systemic roles. Additionally, significant heterogeneity was observed in how IL-33 and ST2 expression was evaluated across studies, including variations in the sample sub-sites, the number of samples assessed, the method applied, and differences in the specific areas and cell types analyzed. This variability prevented the possibility of conducting a meaningful meta-analysis. Furthermore, not all studies examined IL-33 and ST2 expression comprehensively across tumor cells, stromal compartments, and TME, limiting the ability to draw definitive conclusions regarding their broader role in HNSCC pathophysiology. These inconsistencies highlight the need for standardized methodologies to improve comparability and enhance the understanding of IL-33 and ST2 in HNSCC.

## Conclusion

Understanding the interactions among TME, CAFs, and Tregs is crucial for unraveling the complexities of cancer biology. The notion that stromal IL-33 could be a promoter of tumor progression is quite intriguing. The communication between CAFs and IL-33 signaling can influence cancer invasiveness through both paracrine and autocrine pathways, stressing the intricate role of the tumor stroma in shaping malignancy. The connection between overexpressed IL-33 in CAFs and poor clinical outcomes emphasizes the plausible significance of IL-33 as a prognostic marker. The expansion of the Treg population and their enhanced functions mediated by stromal IL-33 in the TME sheds light on the immunosuppressive mechanisms at play. Tregs' role in suppressing local antitumor immune responses concurs with the broader understanding of how the immune system can both fight and inadvertently support tumor growth. The IL-33/ST2 axis seems to be a key player in modulating the TME and influencing the aggressiveness of HNSCC. The discovery of reliable prognostic biomarkers like IL-33 can be potentially useful in HNSCC. Identifying such biomarkers helps in understanding disease progression and individual patient prognosis. This may be pivotal for tailoring treatment strategies, possibly leading to more targeted therapies that could improve outcomes and increase survival rates.

A more comprehensive evaluation of IL-33 and ST2 in both tissue samples and body fluids, combined with the integration of PD-L1 expression analysis, would offer a more holistic perspective on their roles in HNSCC. Addressing the gaps in knowledge, such as the systemic impact of soluble forms, their interaction with immune checkpoint pathways, and the need for standardized methodologies, will pave the way for more targeted and effective therapeutic strategies in HNSCC.

## Data Availability

The original contributions presented in the study are included in the article/Supplementary Material, further inquiries can be directed to the corresponding authors.
