# Supplementary material for: Prognostic significance of IL-33 and ST2 expression in head and neck squamous cell carcinoma: a systematic review
Source: Front Oral Health. 2025 Mar 24;6:1551781. doi: 10.3389/froh.2025.1551781 (PMC11973380; doi:10.3389/froh.2025.1551781)
Supplement: Supplementary file 1 [file Table1.docx]

**Table S1:** Excluded studies with reason

| **Sl No** | **Citation** | **Reason for exclusion** |
| --- | --- | --- |
| 1 | Cui G, Ren J, Xu G, Li Z, Zheng W, Yuan A. Cellular and clinicopathological features of the IL-33/ST2 axis in human esophageal squamous cell carcinomas. *Cancer Cell Int* (2018) 18:203. doi: 10.1186/s12935-018-0700-2. | Squamous cell carcinoma of esophagus |
| 2 | Zare R, Malekzadeh M, Hashemi M, Khademi B, Andishe-Tadbir A. Investigation of IL-33 serum levels in patients with benign and malignant salivary gland tumors. *Cancer Biomark*. (2018) 23(1):61-65. doi: 10.3233/CBM-181309. | Serum estimation of IL-33 in salivary gland tumors |
| 3 | Cui G, Li Z, Ren J, Yuan A. IL-33 in the tumor microenvironment is associated with the accumulation of FoxP3-positive regulatory T cells in human esophageal carcinomas. *Virchows Arch*. (2019) 475(5):579-586. doi: 10.1007/s00428-019-02579-9. | Squamous cell carcinoma of esophagus |
| 4 | Amôr NG, de Oliveira CE, Gasparoto TH, Vilas Boas VG, Perri G, Kaneno R, et al. ST2/IL-33 signaling promotes malignant development of experimental squamous cell carcinoma by decreasing NK cells cytotoxicity and modulating the intratumoral cell infiltrate. *Oncotarget* (2018) 9(56):30894-30904. doi: 10.18632/oncotarget.25768. | Cutaneous squamous cell carcinomas |
| 5 | Yue Y, Lian J, Wang T, Luo C, Yuan Y, Qin G, et al. Interleukin-33-nuclear factor-κB-CCL2 signaling pathway promotes progression of esophageal squamous cell carcinoma by directing regulatory T cells. *Cancer Sci*. (2020) 111(3):795-806. doi: 10.1111/cas.14293. | Squamous cell carcinoma of esophagus |
| 6 | Aarstad HH, Moe SEE, Bruserud Ø, Lybak S, Aarstad HJ, Tvedt THA. The Acute Phase Reaction and Its Prognostic Impact in Patients with Head and Neck Squamous Cell Carcinoma: Single Biomarkers Including C-Reactive Protein Versus Biomarker Profiles. *Biomedicines*. (2020) 8(10):418. doi: 10.3390/biomedicines8100418. | Plasma IL-33 Rα |
| 7 | Mai S, Liu L, Jiang J, Ren P, Diao D, Wang H, Cai K. Oesophageal squamous cell carcinoma-associated IL-33 rewires macrophage polarization towards M2 via activating ornithine decarboxylase. *Cell Prolif*. (2021) 54(2): e12960. doi: 10.1111/cpr.12960. | Squamous cell carcinoma of esophagus |
| 8 | Liu X, Li Z, Ren J, Cui G. IL-33-expressing microvascular endothelial cells in human esophageal squamous cell carcinoma: Implications for pathological features and prognosis. *Microvasc Res.* (2023) 147:104506. doi: 10.1016/j.mvr.2023.104506. | Squamous cell carcinoma of esophagus |
