# Supplementary material for: Prognostic significance of IL-33 and ST2 expression in head and neck squamous cell carcinoma: a systematic review
Source: Front Oral Health. 2025 Mar 24;6:1551781. doi: 10.3389/froh.2025.1551781 (PMC11973380; doi:10.3389/froh.2025.1551781)
Supplement: Supplementary file 2 [file Table2.docx]

**Table S1:** Excluded studies with reason

| **Sl No** | **Author** | **Years** | **Title** | **Reason for exclusion** |
| --- | --- | --- | --- | --- |
| 1 | Cui G et al., ^49^ | 2018 | Cellular and clinicopathological features of the IL-33/ST2 axis in human esophageal squamous cell carcinomas | Squamous cell carcinoma of esophagus |
| 2 | Zare R et al.,^52^ | 2018 | Investigation of IL-33 serum levels in patients with benign and malignant salivary gland tumors | Serum estimation of IL-33 in salivary gland tumors |
| 3 | Cui G et al., ^29^ | 2019 | IL-33 in the tumor microenvironment is associated with the accumulation of FoxP3-positive regulatory T cells in human esophageal carcinomas | Squamous cell carcinoma of esophagus |
| 4 | Amôr NG et al., ^18^ | 2019 | ST2/IL-33 signaling promotes malignant development of experimental squamous cell carcinoma by decreasing NK cells cytotoxicity and modulating the intratumoral cell infiltrate | Cutaneous squamous cell carcinomas |
| 5 | Yue Y et al., ^30^ | 2020 | Interleukin‐33‐nuclear factor‐κB‐CCL2 signaling pathway promotes progression of esophageal squamous cell carcinoma by directing regulatory T cells | Squamous cell carcinoma of esophagus |
| 6 | Aarstad HH et al.,^41^ | 2020 | The Acute Phase Reaction and Its Prognostic Impact in Patients with Head and Neck Squamous Cell Carcinoma: Single Biomarkers Including C-Reactive Protein Versus Biomarker Profiles | Plasma IL-33 Rα |
| 7 | Mai S et al., ^31^ | 2021 | Oesophageal squamous cell carcinoma–associated IL‐33 rewires macrophage polarization towards M2 via activating ornithine decarboxylase | Squamous cell carcinoma of esophagus |
| 8 | Liu X et al., ^53^ | 2023 | IL-33-expressing microvascular endothelial cells in human esophageal squamous cell carcinoma: Implications for pathological features and prognosis | Squamous cell carcinoma of esophagus |
