# Supplementary material for: Prognostic significance of IL-33 and ST2 expression in head and neck squamous cell carcinoma: a systematic review
Source: Front Oral Health. 2025 Mar 24;6:1551781. doi: 10.3389/froh.2025.1551781 (PMC11973380; doi:10.3389/froh.2025.1551781)
Supplement: Supplementary file 4 [file Table4.docx]

| **Scopus - August 10^th^ 2023**  **MeSH/Keywords** | Results |
| --- | --- |
| ( TITLE-ABS-KEY ( "IL-33" )  OR  TITLE-ABS-KEY ( "Interleukin-33" )  OR  TITLE-ABS-KEY ( "ST2" )  OR  TITLE-ABS-KEY ( "Suppression of Tumorigenicity 2" )  AND  TITLE-ABS-KEY ( "Squamous cell carcinoma" )  OR  TITLE-ABS-KEY ( "Head and Neck Squamous cell carcinoma" )  OR  TITLE-ABS-KEY ( "Head and Neck Cancer" ) )  [https://www.scopus.com/results/results.uri?sort=plf-f&src=s&st1="IL-33"&sid=72d1c9323b1617aeee2fc892ac7f8147&sot=b&sdt=b&sl=22&s=(TITLE-ABS-KEY("IL-33")+OR+TITLE-ABS-KEY("Interleukin-33")+OR+TITLE-ABS-KEY("ST2")+OR+TITLE-ABS-KEY("Suppression+of+Tumorigenicity+2")+AND+TITLE-ABS-KEY("Squamous+cell+carcinoma")+OR+TITLE-ABS-KEY("Head+and+Neck+Squamous+cell+carcinoma")+OR+TITLE-ABS-KEY("Head+and+Neck+Cancer"))&origin=searchbasic&editSaveSearch=&yearFrom=Before+1960&yearTo=Present&sessionSearchId=72d1c9323b1617aeee2fc892ac7f8147&limit=10](https://www.scopus.com/results/results.uri?sort=plf-f&src=s&st1=%22IL-33%22&sid=72d1c9323b1617aeee2fc892ac7f8147&sot=b&sdt=b&sl=22&s=%28TITLE-ABS-KEY%28%22IL-33%22%29+OR+TITLE-ABS-KEY%28%22Interleukin-33%22%29+OR+TITLE-ABS-KEY%28%22ST2%22%29+OR+TITLE-ABS-KEY%28%22Suppression+of+Tumorigenicity+2%22%29+AND+TITLE-ABS-KEY%28%22Squamous+cell+carcinoma%22%29+OR+TITLE-ABS-KEY%28%22Head+and+Neck+Squamous+cell+carcinoma%22%29+OR+TITLE-ABS-KEY%28%22Head+and+Neck+Cancer%22%29%29&origin=searchbasic&editSaveSearch=&yearFrom=Before+1960&yearTo=Present&sessionSearchId=72d1c9323b1617aeee2fc892ac7f8147&limit=10) | 81 |
| ( TITLE-ABS-KEY ( "IL-33" )  OR  TITLE-ABS-KEY ( "Interleukin-33" )  OR  TITLE-ABS-KEY ( "ST2" )  OR  TITLE-ABS-KEY ( "Suppression of Tumorigenicity 2" )  AND  TITLE-ABS-KEY ( "Squamous cell carcinoma" )  OR  TITLE-ABS-KEY ( "Head and Neck Squamous cell carcinoma" )  OR  TITLE-ABS-KEY ( "Head and Neck Cancer" ) )  AND  ( LIMIT-TO ( LANGUAGE ,  "English" ) )  <https://www.scopus.com/term/analyzer.uri?sort=plf-f&src=s&sid=72d1c9323b1617aeee2fc892ac7f8147&sot=a&sdt=a&cluster=scolang%2c%22English%22%2ct&sl=279&s=%28TITLE-ABS-KEY%28%22IL-33%22%29+OR+TITLE-ABS-KEY%28%22Interleukin-33%22%29+OR+TITLE-ABS-KEY%28%22ST2%22%29+OR+TITLE-ABS-KEY%28%22Suppression+of+Tumorigenicity+2%22%29+AND+TITLE-ABS-KEY%28%22Squamous+cell+carcinoma%22%29+OR+TITLE-ABS-KEY%28%22Head+and+Neck+Squamous+cell+carcinoma%22%29+OR+TITLE-ABS-KEY%28%22Head+and+Neck+Cancer%22%29%29&origin=resultslist&count=10&analyzeResults=Analyze+results> | 81 |
| ( TITLE-ABS-KEY ( "IL-33" )  OR  TITLE-ABS-KEY ( "Interleukin-33" )  OR  TITLE-ABS-KEY ( "ST2" )  OR  TITLE-ABS-KEY ( "Suppression of Tumorigenicity 2" )  AND  TITLE-ABS-KEY ( "Squamous cell carcinoma" )  OR  TITLE-ABS-KEY ( "Head and Neck Squamous cell carcinoma" )  OR  TITLE-ABS-KEY ( "Head and Neck Cancer" ) )  AND  ( LIMIT-TO ( LANGUAGE ,  "English" ) )  AND  ( LIMIT-TO ( DOCTYPE ,  "ar" ) )  <https://www.scopus.com/results/results.uri?sort=plf-f&src=s&st1=%22IL-33%22&sid=72d1c9323b1617aeee2fc892ac7f8147&sot=b&sdt=cl&sl=22&s=%28TITLE-ABS-KEY%28%22IL-33%22%29+OR+TITLE-ABS-KEY%28%22Interleukin-33%22%29+OR+TITLE-ABS-KEY%28%22ST2%22%29+OR+TITLE-ABS-KEY%28%22Suppression+of+Tumorigenicity+2%22%29+AND+TITLE-ABS-KEY%28%22Squamous+cell+carcinoma%22%29+OR+TITLE-ABS-KEY%28%22Head+and+Neck+Squamous+cell+carcinoma%22%29+OR+TITLE-ABS-KEY%28%22Head+and+Neck+Cancer%22%29%29&origin=resultslist&editSaveSearch=&yearFrom=Before+1960&yearTo=Present&sessionSearchId=72d1c9323b1617aeee2fc892ac7f8147&limit=10&cluster=scolang%2C%22English%22%2Ct%2Bscosubtype%2C%22ar%22%2Ct> | 49 |
| ( TITLE-ABS-KEY ( "IL-33" ) OR TITLE-ABS-KEY ( "Interleukin-33" ) OR TITLE-ABS-KEY ( "ST2" ) OR TITLE-ABS-KEY ( "Suppression of Tumorigenicity 2" ) AND TITLE-ABS-KEY ( "Squamous cell carcinoma" ) OR TITLE-ABS-KEY ( "Head and Neck Squamous cell carcinoma" ) OR TITLE-ABS-KEY ( "Head and Neck Cancer" ) ) AND PUBYEAR > 2012 AND PUBYEAR < 2024 AND ( LIMIT-TO ( LANGUAGE , "English" ) ) AND ( LIMIT-TO ( DOCTYPE , "ar" ) )  <https://www.scopus.com/results/results.uri?sort=plf-f&src=s&st1=%22IL-33%22&sid=72d1c9323b1617aeee2fc892ac7f8147&sot=b&sdt=cl&sl=22&s=%28TITLE-ABS-KEY%28%22IL-33%22%29+OR+TITLE-ABS-KEY%28%22Interleukin-33%22%29+OR+TITLE-ABS-KEY%28%22ST2%22%29+OR+TITLE-ABS-KEY%28%22Suppression+of+Tumorigenicity+2%22%29+AND+TITLE-ABS-KEY%28%22Squamous+cell+carcinoma%22%29+OR+TITLE-ABS-KEY%28%22Head+and+Neck+Squamous+cell+carcinoma%22%29+OR+TITLE-ABS-KEY%28%22Head+and+Neck+Cancer%22%29%29&origin=resultslist&editSaveSearch=&yearFrom=2000&yearTo=2023&sessionSearchId=72d1c9323b1617aeee2fc892ac7f8147&limit=10&cluster=scolang%2C%22English%22%2Ct%2Bscosubtype%2C%22ar%22%2Ct> | 41 |
| Title and abstract screening | 17 |
| Full text with inclusion and exclusion criteria | 9 |
| Excluded studies | 8 |
