# Supplementary material for: Prognostic significance of IL-33 and ST2 expression in head and neck squamous cell carcinoma: a systematic review
Source: Front Oral Health. 2025 Mar 24;6:1551781. doi: 10.3389/froh.2025.1551781 (PMC11973380; doi:10.3389/froh.2025.1551781)
Supplement: Supplementary file 6 [file Table6.docx]

| **Web of Science** | | **10^th^ Aug 2023** |
| --- | --- | --- |
| **Search strategy** | **MeSH/Keywords** | Results |
| 1 | <https://www.webofscience.com/wos/woscc/summary/fc17612e-5d4b-494c-bf49-7b6e7b3eecd0-9a586319/relevance/1>  ((((((ALL=("IL-33")) OR ALL=("Interleukin-33")) OR ALL=("ST2")) OR ALL=("Suppression of Tumorigenicity 2")) AND ALL=("Squamous cell carcinoma")) OR ALL=("Head and Neck Squamous cell carcinoma")) OR ALL=("Head and Neck cancer") | 48,381 |
| 2 | <https://www.webofscience.com/wos/woscc/summary/5fffb70d-598a-41e0-9adc-a34812bb5ee4-9a841612/relevance/1>  **((((((ALL=("IL-33")) OR ALL=("Interleukin-33")) OR ALL=("ST2")) OR ALL=("Suppression of Tumorigenicity 2")) AND ALL=("Squamous cell carcinoma")) OR ALL=("Head and Neck Squamous cell carcinoma")) OR ALL=("Head and Neck cancer")** and **English** (Languages) | 47,404 |
| 3 | <https://www.webofscience.com/wos/woscc/summary/06b4116b-84dd-41be-88e8-5df90860576c-9a5b76b6/relevance/1>  **((((((ALL=("IL-33")) OR ALL=("Interleukin-33")) OR ALL=("ST2")) OR ALL=("Suppression of Tumorigenicity 2")) AND ALL=("Squamous cell carcinoma")) OR ALL=("Head and Neck Squamous cell carcinoma")) OR ALL=("Head and Neck cancer")** and **Article** (Document Types) and **English** (Languages) | 31,417 |
| 4 | **((((((ALL=("IL-33")) OR ALL=("Interleukin-33")) OR ALL=("ST2")) OR ALL=("Suppression of Tumorigenicity 2")) AND ALL=("Squamous cell carcinoma")) OR ALL=("Head and Neck Squamous cell carcinoma")) OR ALL=("Head and Neck cancer")** and **English** (Languages) and **Article** (Document Types) and **Proceeding Paper** or **Early Access** or **Book Chapters** or **Retracted Publication** or **Data Paper** (Exclude – Document Types) <https://www.webofscience.com/wos/woscc/summary/b1f198e3-f7d7-4c81-85d5-970f850dba5b-9c94c310/relevance/1> | 28,741 |
| 5 | **((((((ALL=("IL-33")) OR ALL=("Interleukin-33")) OR ALL=("ST2")) OR ALL=("Suppression of Tumorigenicity 2")) AND ALL=("Squamous cell carcinoma")) OR ALL=("Head and Neck Squamous cell carcinoma")) OR ALL=("Head and Neck cancer")** and **English** (Languages) and **Article** (Document Types) and **Proceeding Paper** or **Book Chapters** or **Early Access** or **Data Paper** or **Retracted Publication** (Exclude – Document Types) and **2013** or **2014** or **2015** or **2016** or **2017** or **2018** or **2019** or **2020** or **2021** or **2022** or **2023** (Publication Years)  <https://www.webofscience.com/wos/woscc/summary/265c98f1-6836-41b2-8e24-953b58425879-9c95eb96/relevance/1> | 19,871 |
| 6 | **ALL=("Head and Neck Squamous cell carcinoma")) OR ALL=("Head and Neck cancer")** and **English** (Languages) and **Article** (Document Types) and **Proceeding Paper** or **Book Chapters** or **Early Access** or **Data Paper** or **Retracted Publication** (Exclude – Document Types) and **2013** or **2014** or **2015** or **2016** or **2017** or **2018** or **2019** or **2020** or **2021** or **2022** or **2023** (Publication Years) and **Oncology** or **Otorhinolaryngology** or **Biochemistry Molecular Biology** or **Pathology** (Research Areas)  <https://www.webofscience.com/wos/woscc/summary/44bf45ec-68ee-4da2-8107-104a23c2101d-9c965bde/relevance/1> | 12,540 |
| 7 | Title and abstract screening | 9 |
| 8 | Full text with inclusion and exclusion criteria | 6 |
| 9 | Excluded studies | 3 |
